# Oncogenic functions of protein kinase D2 and D3 in regulating multiple cancer‐related pathways in breast cancer

**DOI:** 10.1002/cam4.1938

**Published:** 2019-01-16

**Authors:** Yan Liu, Jian Li, Zhifang Ma, Jun Zhang, Yuzhi Wang, Zhenghong Yu, Xue Lin, Zhi Xu, Qian Su, Li An, Yehui Zhou, Xinxing Ma, Yiwen Yang, Feifei Wang, Qingfei Chen, Yunchao Zhang, Jilinlin Wang, Huilin Zheng, Aihua Shi, Shuang Yu, Jingzhong Zhang, Weiyong Zhao, Liming Chen

**Affiliations:** ^1^ Jiangsu Key Laboratory for Molecular and Medical Biotechnology, College of Life Science Nanjing Normal University Nanjing China; ^2^ The Key Laboratory of Developmental Genes and Human Disease, Ministry of Education, Institute of Life Sciences Southeast University Nanjing China; ^3^ The Key Laboratory of Bio‐Medical Diagnostics, Suzhou Institute of Biomedical Engineering and Technology Chinese Academy of Sciences Suzhou China; ^4^ Changchun Institute of Optics, Fine Mechanics and Physics Chinese Academy of Sciences Changchun China; ^5^ Department of Medical Oncology, Jinling Hospital Medical School of Nanjing University Nanjing China; ^6^ Department of Bioinformatics Nanjing Medical University Nanjing China; ^7^ Department of Oncology Tongren Hospital Shanghai Jiao Tong University School of Medicine Shanghai China; ^8^ The First Affiliated Hospital of Soochow University Soochow University Suzhou China

**Keywords:** breast cancer, phosphoproteome analysis, PKD2, PKD3, transcriptome analysis

## Abstract

Protein Kinase D (PKD) family contains PKD1, PKD2, and PKD3 in human. Compared to consistent tumor‐suppressive functions of PKD1 in breast cancer, how PKD2/3 functions in breast cancer are not fully understood. In the current study, we found that PKD2 and PKD3 but not PKD1 were preferentially overexpressed in breast cancer and involved in regulating cell proliferation and metastasis. Integrated phosphoproteome, transcriptome, and interactome showed that PKD2 was associated with multiple cancer‐related pathways, including adherent junction, regulation of actin cytoskeleton, and cell cycle‐related pathways. ELAVL1 was identified as a common hub‐node in networks of PKD2/3‐regulated phosphoproteins and genes. Silencing *ELAVL1* inhibited breast cancer growth in vitro and in vivo. Direct interaction between ELAVL1 and PKD2 or PKD3 was demonstrated. Suppression of *PKD2* led to ELAVL1 translocation from the cytoplasm to the nucleus without significant affecting ELAVL1 expression. Taken together, we characterized the oncogenic functions of PKD2/3 in breast cancer and their association with cancer‐related pathways, which shed lights on the oncogenic roles and mechanisms of PKDs in breast cancer.

## 1 INTRODUCTION

Breast cancer is a major cancer type in women. Protein Kinase D (PKD) family, a family of serine/threonine kinases, includes three family members of PKD1, PKD2, and PKD3. PKDs are involved in a large variety of cellular and physiological processes which are crucial for tumorigenesis and tumor progression, including cell proliferation, migration, invasion, and angiogenesis.^1^, ^2^, ^3^ The three isoforms have high sequence homology as the highly conserved N‐terminal regulatory domain containing two cysteine‐rich DAG‐binding C1 domains and an auto‐inhibitory pleckstrin homology (PH) domain. Although PKD1 and PKD2 share ~85% overall homology, particularly in their catalytic domain and C1 domains, deletion of the C1 domains in PKD1 leads to increased kinase activity,^4^ where that in PKD2 decreased kinase activity.^5^ For PKD3, comparing to PKD1/2, it lacks PDZ (PSD‐95/Disks large/ZO‐1) binding motif^6^ and a Src family kinase phosphorylation motif.^7^ These structural and functional differences suggest differentially regulated signaling pathways in cellular biological activities.

Dysregulated PKDs have been found in many cancers, where the three isoforms are reported to be either tumor suppressor or oncogene depending on cellular context. In gastric cancers, PKD1 functions as a tumor suppressor via epigenetic inactivation.^8^ However, in skin and pancreatic cancers, PKD1 plays important roles in tumor‐promoting processes such as increasing DNA replication, inhibiting apoptosis, promoting proliferation through positively regulating ERK, MAPK pathways.^9^, ^10^, ^11^ PKD2 was reported as a tumor‐promoting protein in prostate, pancreas, stomach, and glioblastoma cancer via inducting angiogenesis, inhibiting apoptosis by activating NF‐kB, MMP signal pathways.^12^, ^13^, ^14^, ^15^ PKD3 showed pro‐oncogenic properties in prostate and skin cancer.^12^, ^16^

In the normal breast ductal epithelial cells, PKD1 is the major isoform highly expressed, where PKD2 and PKD3 are expressed at moderate levels.^17^, ^18^ Loss of PKD1 was frequently found in breast cancer through epigenetic silencing to enhanced breast cancer cells invasion, metastasis, and tumor progression. PKD3 was identified to be a main oncogenic PKD isoform in breast cancer,^19^, ^20^ participating in breast tumor growth and metastasis.^21^, ^22^ While the function of PKD2 in breast cancer is not clear. Silencing *PKD2* significantly decreased cell proliferation of HCC1806 breast cancer cells,^23^ while a report claimed that *PKD2* expression level was reduced in triple‐negative breast cancer.^22^ Whether and how PKD2 functions in breast cancer is still obscure.

There is a growing body of evidence supporting the significance of PKD family members in breast cancer development. However, the precise mechanisms and networks of these kinases contributing to tumorigenesis and invasiveness are still unclear. To this end, we investigated the oncogenic functions of PKD2 and PKD3 in vitro and in vivo, as well as their associated cancer‐related pathways through integrated omics study.

## 2 MATERIALS AND METHODS

### 2.1 Cell culture

MDA‐MB‐468 and MDA‐MB‐231 cells were maintained in Dulbecco's modified essential medium (Life Technologies, Carlsbad, CA, USA) supplemented with 1% penicillin‐streptomycin solution (Life Technologies) and 10% fetal bovine serum (HyClone, Logan, UT, USA). T47D cells were cultured in RPMI 1640 medium (Gibco, Grand Island, NY, USA) supplemented with 1% penicillin‐streptomycin solution and 10% fetal bovine serum. MCF10A cells were maintained in DMEM/F12 media with 10% horse serum, 100 ng/mL cholera toxin, 20 ng/mL epidermal growth factor (EGF), 500 ng/mL hydrocortisone, 0.01 mg/mL insulin and 1% penicillin‐streptomycin solution. In the current study, all cell lines used were all origin from ATCC and used within 6 months after authenticated via the short tandem repeat (STR) typing.

### 2.2 RNA interference

ON‐TARGET plus siRNA targeting PKD2 (PKD2 siRNA‐1:5′‐CGACCAACAGAUACUAUAA‐3′, PKD2 siRNA‐2:5′‐CAAUGGAGAUGUGCCGAUG‐3′), PKD3 (PKD3 siRNA‐1:5′‐GGAUGUGGCUAUUAAAGUA‐3′, PKD3 siRNA‐2:5′‐GCUGGGAAAUACAUGCAUA‐3′) or the non‐targeting control siRNA (5′‐UGGUUUACAUGUCGACUAA‐3′) were purchased from Dharmacon and transfected into breast cancer cells using Lipofectamine RNAi MAX (Invitrogen, Carlsbad, CA, USA) according to the manufacturer's instructions. Final concentration of siRNA is 10 nM. The cells were incubated at 37°C in a CO2 incubator for 72 hours and then subjected to protein or RNA extraction.

### 2.3 Generation of stable cell lines

MDA‐MB‐231 with stable depletion of PKD2 was generated using lentivirus shRNA system. PL‐CMV‐GFP‐WPRE‐U3‐NHE1 was used to generate PKD2 lentiviral shRNA constructs with following primers: 5′CTAGCCAAAAACGACCAACAGATACTATAATCTCTTGAATTATAGTATCTGTTGGTCG 3′ and 5′CTAGCCGACCAACAGATACTATAATTCAAGAGATTATAGTATCTGTTGGTCGTTTTTG 3′ according to the manufacturer's instructions.

MDA‐MB‐231 with stable depletion of ELAVL1 was generated using lentivirus shRNA system. pLKO.1‐TRC was used to generate ELAVL1 lentiviral shRNA constructs with following primers: ELAVL1 shRNA‐1 5′CCGGGATCAGACTACAGGTTTGTCTCGAGACAAACCTGTAGTCTGATCTTTTTG 3′ and 5′AATTCAAAAAGATCAGACTACAGGTTTGTCTCGAGACAAACCTGTAGTCTGATC 3′,ELAVL1 shRNA‐2 5′CCGGGAGGCAATTACCAGTTTCACTCGAGTGAAACTGGTAATTGCCTCTTTTTG 3′ and 5′AATTCAAAAAGAGGCAATTACCAGTTTCACTCGAGTGAAACTGGTAATTGCCTC 3′.

### 2.4 Western blot

Rabbit antibodies against PKD1, PKD2, PKD3, Ki67, caspase9, and desmin were purchased from Cell Signaling Technology (Beverly, MA, USA). Anti‐rabbit secondary antibody, anti‐mouse secondary antibody, and β‐actin antibody were purchased from Kangchen, anti‐ELAVL1 antibody was purchased from Absci. N‐Ras antibody was purchased from Santa Cruz Biotechnology (Dallas, TX, USA). Western blot was carried out following the standard procedure. Briefly, protein lysates were separated by SDS‐PAGE, transferred to PVDF membranes, and immunoblotted with the respective antibodies as indicated above and in the figures. Blots were developed with SuperSignal West Femto Maximum Sensitivity Substrate (Pierce/Thermo Scientific, Rockford, IL, USA).

### 2.5 Cell proliferation, cell cycle assay, cell apoptosis, and cell migration assay

Cell proliferation was measured with the CCK‐8 kit (Dojindo Laboratories, Kumamoto, Japan) according to the protocol recommended by the manufacturer. For cell cycle analysis, unsynchronized cells were harvested by trypsinization and fixed with 70% ethanol. Cells were then stained with propidium iodide for total DNA content and the cell cycle distribution was then analyzed using a BD FACSCalibur flow cytometry (Becton Dickinson). For the cellular apoptosis assay, cells were stained using Annexin‐V/Dead Cell Apoptosis Kit (Invitrogen) as per the manufacturer's recommendations and analyzed on a BD FACSCalibur flow cytometry (BD Biosciences, Franklin Lakes, NJ, USA). For cell migration assay, monolayers of cells were grown on 6 well plates before a cell‐free region was created using a 10 μL pipette tip. Scratch wound width was measured using a graticule at 0 and 12 hours post treatment. Representative images were taken at these time points using a Carl Zeiss Microscopy at ×5 magnification.

### 2.6 Immunofluorescence

MDA‐MB‐231s were grown on glass slides. CTRL and PKD2 siRNAs were added into the well and cultured for 72 hours. Prior to staining, MDA‐MB‐231 cells were washed with PBS, the cells were treated with 4% PFA for 30 minutes, then permeabilized and blocked with 0.1% Triton X‐100 in 1% bovine serine albumin for 1 hour at room temperature. Rabbit anti‐ELAVL1 (Absci, Hangzhou, China) was used as primary antibodies, and Alexa Fluor 488‐conjugated secondary antibody was used to detect fluorescence. The nuclei were stained with DAPI (Solarbio, Shanghai, China). Representative images were captured using the Leica microscope .

### 2.7 Co‐immunoprecipitation

Co‐immunoprecipitation (Co‐IP) was performed using anti‐PKD2, anti‐PKD3 antibody (Cell Signaling Technology), anti‐ELAVL1 (Absci, Hangzhou, China), and Dynabeads Protein G (Invitrogen) according to manufacturer's instruction. In brief, cell lysates were incubated with anti‐ antibody‐conjugated beads at 4°C for 2 hours. Then, the beads were washed extensively and boiled in SDS loading buffer. MS analysis and western blot were used to study the immunoprecipitated proteins.

### 2.8 PKD2‐ and PKD2&3‐regulated phosphoproteome analysis using iTRAQ

Cells were prepared using Ready Prep Protein Extraction kit (Bio‐RAD, Hercules, CA, USA). Extracted protein concentration was determined by BioSpec‐nano (Shimadzu Biotech, Kyoto, Japan). Approximately 4 mg of protein/sample was used for quantitative phosphoproteomic profiling.

Each protein sample was reduced and alkylated and digested with trypsin (Promega, Beijing, China). Following tryptic digestion, peptide samples were desalted using MonoTip C18 (Shimadzu Biotech). The eluted peptides were dried in a SpeedVac and then labeled with iTRAQ8‐plex reagents according to the manufacturer's instructions.

Phosphopeptide enrichment was performed using TitanspherePhos‐TiO kit (Shimadzu Biotech) according to the manufacturer's instructions. Elution of phosphopeptides was combined, acidified with 100 mL of 2.5% trifluoroacetic acid, desalted with the MonoTip C18 (Shimadzu Biotech) and resuspended in 0.1% formic acid. Samples were analyzed using Prominence nanoflow LC system (Shimadzu Biotech) connected to an LCMS‐IT‐TOF mass spectrometer (Shimadzu Biotech). The detected fragments were searched with ProteinLayer software using Swiss‐Prot human database and the phosphorylation sites were determined using a PTM Finder Software (Shimadzu Biotech).

Abundance ratios between samples were quantified by LabSolution Software (Shimadzu Biotech) via the quantification of iTRAQ labeled peptides with a synthetic peptide corresponding to the residues between 14 and 38 (“TQCPDDSTCCELPTGK”) of mouse Granulin3 labeled with [d0]/[d6]‐DMPITC used as an internal standard for quantification (BioworldInc, Nanjing, China). To minimize contaminating near isobaric ions, only the peptides with isolation specificity more than 75% were quantified.

### 2.9 Transcriptome investigation using Affymetrix microarray

Total RNA from cells was isolated using the RNeasy kit (Qiagen, Hilden, Germany) according to the manufacturer's instruction. Total RNA was eluted in a final volume of 30 μl (ddH2O) and stored at −80°C until further processing. The GeneChip 3’IVT Express Kit (Cat#901229, Affymetrix, Santa Clara, CA, US) was used to synthesize double‐stranded cDNA and produce biotin‐labeled cRNA from 500 ng of total RNA. After fragmentation, 10 μg of cRNA were hybridized at 45°C for 16 h to Affymetrix HG_U133 Plus 2.0 oligonucleotide arrays containing probes to more than 47,000 transcripts. Array hybridization and wash was performed using GeneChip® Hybridization, Wash and Stain Kit (Cat#900720, Affymetrix, Santa Clara, CA, US) in Hybridization Oven 645(Cat#00‐0331‐220 V, Affymetrix, Santa Clara, CA, US), and Fluidics Station 450 (Cat#00‐0079, Affymetrix, Santa Clara, CA, US) followed the manufacturer's instructions. Slides were scanned by GeneChip® Scanner 3000 (Cat#00‐00212, Affymetrix, Santa Clara, CA, US) and Command Console Software 3.1 (Affymetrix, Santa Clara, CA, US) with default settings.

### 2.10 Data preprocessing of Affymetrix microarray gene expression

The raw data of the expression array, CEL files, were input for a series of analyses including quality control, data preprocessing, and identifying differential expressed genes. Briefly, the array was annotated with hgu133plus2 homo sapiens ensemble genes.^24^ The GCRMA was applied for normalization.^25^ Then, a t‐test based approach^26^ was used for calling differentially expressed genes between sample pair‐comparisons.

### 2.11 Real‐time RT‐qPCR

Total RNA was extracted from breast cancer cells using the RNeasy kit (Qiagen) and the synthesized cDNA was performed using PrimeScript RT reagent kit (TaKaRa, Otsu, Shiga, Japan). Quantitative PCR reactions were performed using SYBRPremix Ex Taq (TaKaRa) in a Bio‐Rad CFX96 Real‐Time PCR System (Bio‐Rad). β‐actin was used as an internal control. The real‐time PCR primer sequences are listed in Table S1.

### 2.12 In vivo mouse model

Female athymic STOCK‐Foxn1^nu^/Nju 4‐week‐old mice were obtained from Model Animal Research Center of Nanjing University. The cells (5 × 10^6^cells) were subcutaneously injected into the armpit of mice respectively. The tumor size of PKD2 silenced and control group was measured for 4 weeks. After inoculation of ELAVL1 silenced and control cells for 3 weeks, the mice were killed to take out of tumor, using a caliper and tumor volume was calculated by the following formula: Volume = 0.5 × Length × Wideth^2^. All of the experiments were conducted in accordance with the instructional standard guideline of Southeast University for animal experiments.

### 2.13 Meta‐analysis of gene expression of breast cancers from public database

The standardized data yielded a dataset of 1888 breast cancer tumors with distant metastasis‐free survival time information. We applied Array Generation based gene Centering (AGC) normalization method to perform normalize all samples.^27^ Briefly, we assume that μi,k = μk (i is different arrays, k is a gene, μ is the mean expression of the gene). Then, we assume that the minimum and the maximum estimates for the gene value are reached and the range of the gene k should approximately be [αk, bk], where αk is the lowest 2% value and bk is the largest 2% value of gene k. If the new centered value exceeds the range, the difference is diminished toward the range limits with coefficient c, 0 ≤ c ≤ 1. Here, the coefficient is set to c = 1/5 in order to diminish the greatest and smallest values.^28^

### 2.14 Breast cancer samples

Breast cancer samples were obtained from the Jinling Hospital. The breast cancer samples were instantly frozen in liquid nitrogen after excision. H&E stained frozen sections were prepared from each breast cancer sample to confirm benignity or malignancy and to obtain information about histopathological grade and histological subtype. All the breast cancer samples were analyzed anonymously and were acquired with the written consent of the patients. This study was performed with the approval of the medical ethics committee of Southeast University.

## 3 RESULTS

### 3.1 Elevated PKD2/3 expression in breast cancer

**Figure 1:**
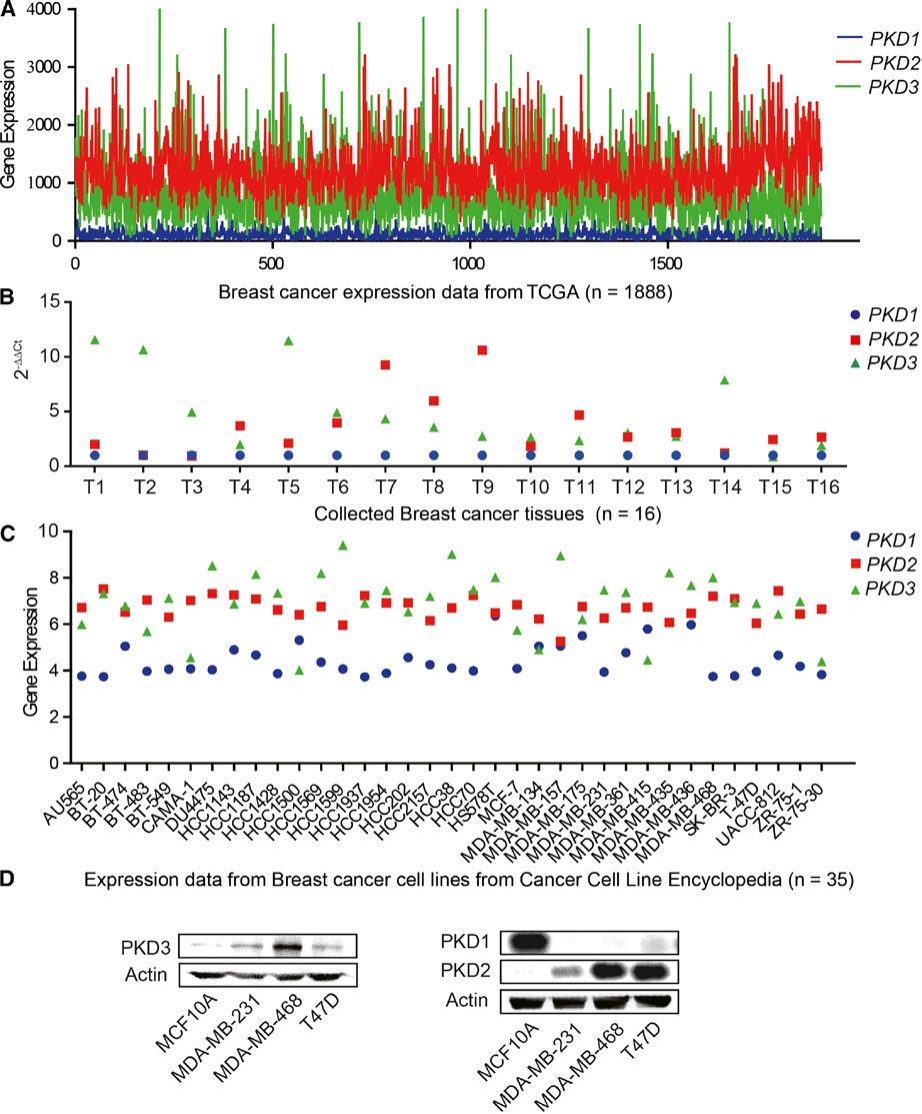
PKDs expression analysis in breast cancer. A, Expression analysis of PKDs in 1888 breast cancer samples from TCGA. B, Expression analysis of PKDs in 16 collected breast cancer tissues using RT‐qPCR. C, Expression analysis of PKDs in analysis expression data of breast cancer cell lines. D, Expression analysis of PKDs in non‐cancerous breast cell line MCF10A and breast cancer cell lines, MDA‐MB‐231, MDA‐MB‐468, and T47D, using western blot

*PKDs* mRNA expression was analyzed in 1888 breast cancer from TCGA dataset. Both *PKD2* and *PKD3* were preferentially expressed in breast cancer compared to *PKD1* (Figure 1A, Figure S1 and Table S2). These findings were further validated in 16 breast cancer tissues by RT‐qPCR. Consistently, both *PKD2* and *PKD3* were preferentially expressed in most of the breast cancer tissues (13 out of the 16 breast cancer tissues) (Figure 1B, Figure S1, and Table S3). Compared to *PKD1, PKD2* and *PKD3* were expressed at higher levels in the invasive breast cancer cell lines through analyzing the gene expression data from Cancer Cell Line Encyclopedia project and NCI60 Cell Line project (Figure 1C, Figure S1 and Table S4). PKD2 and PKD3 were found overexpressed in MDA‐MB‐231, MDA‐MB‐468, and T47D, but not in non‐cancerous breast cell line MCF10A, whereas PKD1 was only expressed in MCF10A (Figure 1D).

### 3.2 PKD2 exerts oncogenic functions in breast cancer

**Figure 2:**
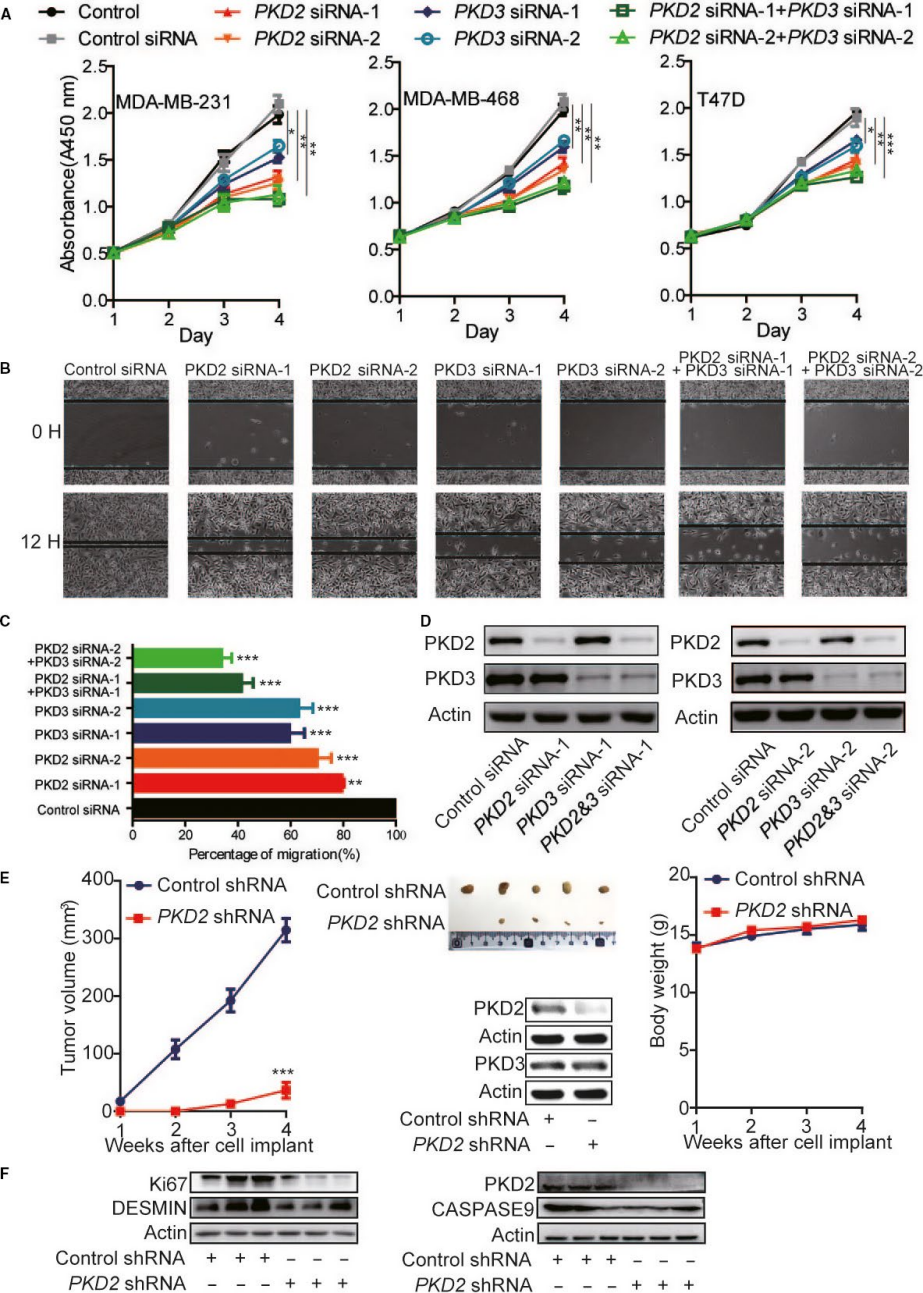
Oncogenic functions of PKD2 in breast cancer. A, Inhibition of proliferation of breast cancer cell lines upon silencing *PKD2* or *PKD3* or both *PKD2* and *PKD3*. B‐C, Inhibition of migration of breast cancer cell lines upon silencing *PKD2* or *PKD3* or both *PKD2* and *PKD3*. D, A representative western blot of MDA‐MB‐231 cells shows that PKD2 and PKD3 were specifically and efficiently silenced by *PKD2* siRNAs and *PKD3* siRNAs, respectively. E, Silencing *PKD2* by *PKD2* shRNA inhibited breast tumor growth in xenograft mouse model using MDA‐MB‐231. Western blot indicated that silencing *PKD2* with shRNA can specifically and effectively knockdown *PKD2* not *PKD3* in MDA‐MB‐231. We used 5 xenograft mice for control shRNA and 5 xenograft mice for *PKD2* shRNA. Palpable tumor growth across time was measured every one week from the time tumor was palpable until the animals were sacrificed (week 4). Tumor images at the end point (week 4) were shown and palpable tumor volumes were measured by width and length with a Vernier caliper and calculated by formula *Volume = (Length × Width × Width)/2*. Error bars represent mean ± SD. The *t*‐test was used for calculation of *P* value. “*,” “**,” and “***” stand for *P *< 0.05, *P *< 0.01, and *P *< 0.001, respectively. F, Western blot detected the protein level of Ki67, DESMIN, and CASPASE9 upon *PKD2* silenced by *PKD2* shRNA

To investigate the oncogenic roles of PKD2 and PKD3, cell proliferation and migration were examined in breast cancer cells through manipulating PKD2 and PKD3 expression. As shown in Figure 2A‐D, silencing *PKD2* or *PKD3 *expression in MDA‐MB‐231 inhibited cell proliferation and migration. Furthermore, silencing both of them showed synergistic inhibitory effect on cell proliferation and migration (Figure 2A‐D). In xenograft mouse models, silencing *PKD2* in MDA‐MB‐231 using shRNA led to significant reduction of tumor volume (Figure 2E). Western blot showed that decreased Ki67 and desmin but not caspase9 were found in tumors with abrogated PKD2 when comparing to controls (Figure 2F).

### 3.3 Phosphoproteome analysis of PKD2 in breast cancer

**Figure 3:**
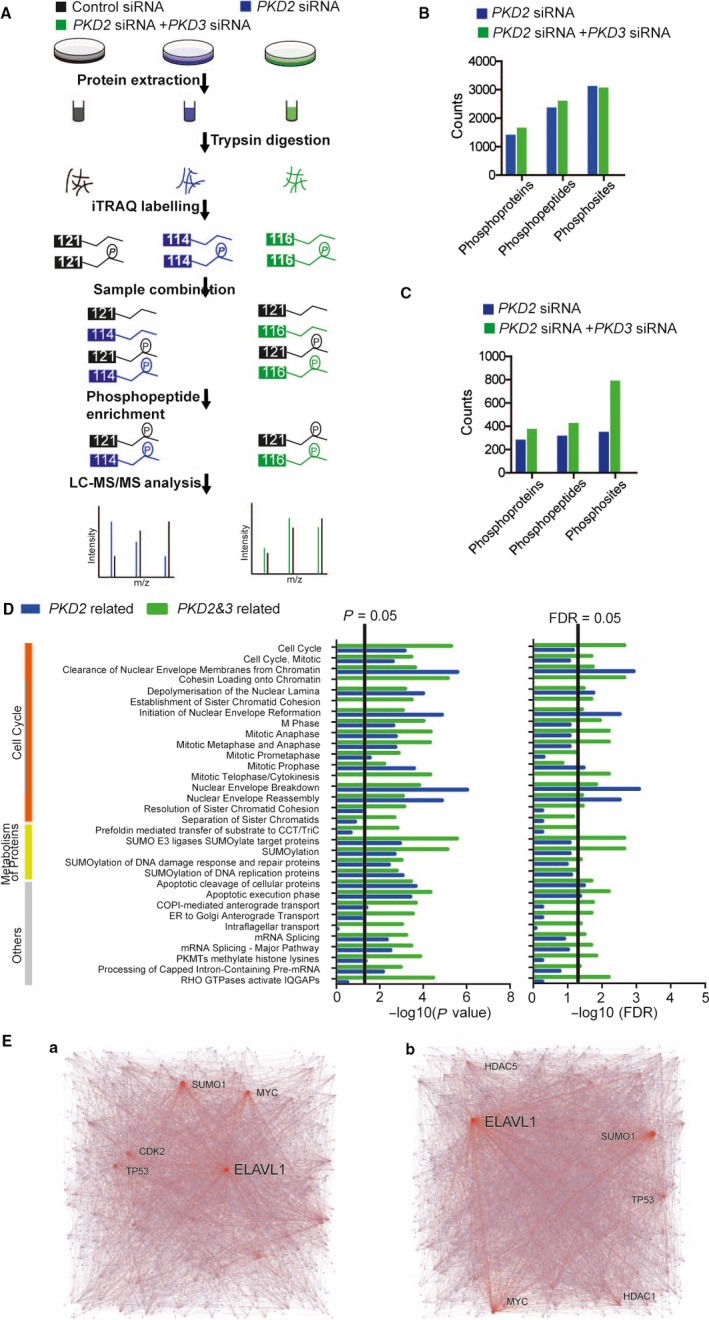
Phosphoproteome analysis of PKD2. A, Flowchart of phosphoproteome analysis. B, Analysis of identified phosphoproteins, phosphopeptides and phosphosites from phosphoproteome. C, Analysis of identified PKD2‐ and PKD2&3‐regulated phosphoproteins, phosphopeptides, and phosphosites. D, Enriched pathway analysis of PKD2‐ and PKD2&3‐regulated phosphoproteins using reactome. E, Network analysis of PKD2‐ and PKD2&3‐regulated phosphoproteins with labeled hub‐nodes

Since PKD2 is a Serine/Threonine kinase, phosphoproteome analysis was used to investigate the potential substrates of PKD2. The iTRAQ labeling phosphoproteome of MDA‐MB‐231 cells transfected with control siRNA, *PKD2* siRNA or with both *PKD2* siRNA and *PKD3* siRNA (*PKD2&3 *siRNA) were used for comparisons (Figure 3A). In total, 3621 phosphopeptides matching 4528 phosphosites of 1999 phosphoproteins were identified with *P*‐value <0.005 (Table S5). Among them, 2334 phosphopeptides of 1370 phosphoproteins were identified upon *PKD2* silencing, while 2565 phosphopeptides and 1623 phosphoproteins were identified upon silencing *PKD2*&*3* (Figure 3B and Table S6). In the phosphoproteome, 308 phosphopeptides of 273 phosphoproteins were identified as PKD2 regulated (sample/control with fold change >1.5), while 418 phosphopeptides and 366 phosphoproteins were identified to be PKD2&3 regulated (Figure 3C and Table S7). Previously, we have reported 308 phosphopeptides and 270 phosphoproteins to be PKD3 regulated.^20^ These findings suggested some proteins could be commonly regulated by both PKD2 and PKD3 in breast cancer. The reactome profiles of PKD2‐, PKD3‐, and PKD2&3‐regulated phosphoproteins were similar (Figure S2A‐B).^20^ In addition, a total of 32 enriched pathway (FDR <0.05 and *P* < 0.05) was identified through analysis of the enriched pathways in the reactomes of PKD2‐ and PKD2&3‐regulated phosphoproteins. Among them, 29 pathways were commonly regulated between PKD2 and PKD2&3 (Figure 3D and Table S8). In these 29 commonly enriched pathways, 17 pathways were involved in cell cycle. Furthermore, network analysis revealed that ELAV like RNA Binding Protein 1 (ELAVL1) was one of the common hub‐nodes between the networks of PKD2‐regulated phosphoproteins and PKD2&3‐regulated phosphoproteins (Figure 3Ea‐b and Figure S3 and Table S9). As for the network of PKD3‐regulated phosphoproteins,^20^ 22 out of these 29 pathways were enriched, and ELAVL1 was identified as a central node. Taken together, the overall consistence between PKD2‐, PKD3‐, and PKD2&3‐regulated phosphoproteins in their reactome profiles, enriched pathways and hub‐nodes indicated the underlying mechanisms of the oncogenic roles of PKD2 in breast cancer.

### 3.4 Transcriptome analysis of PKD2 in breast cancer

**Figure 4:**
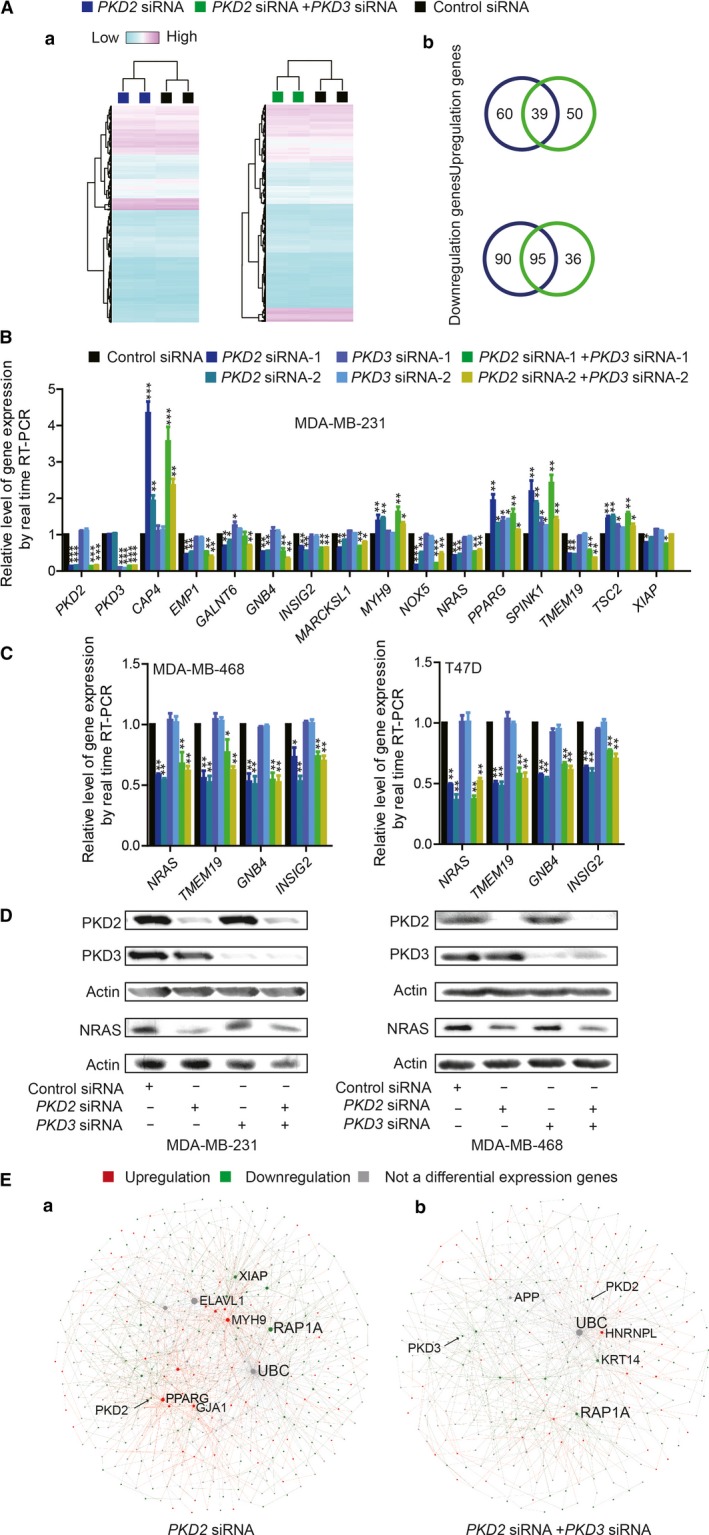
Transcriptome analysis of PKD2. (A‐a) Heatmap, (A‐b) Venn diagram of PKD2‐ and PKD2&3‐regulated genes. (B) Validations of sixteen PKD2‐ and PKD2&3‐regulated genes in MDA‐MB‐231 using RT‐qPCR. (C) RT‐qPCR and (D) Western blot validation of selected four PKD2‐ and PKD2&3‐regulated genes. (E) Network analysis of PKD2‐ and PKD2&3‐regulated genes with labeled hub‐nodes and differential expression information. RT‐qPCR experiments were carried out in triplicates. Error bars represent mean ± SD. The *t*‐test was used for calculation of *P* value. “*,” “**,” and “***” stand for *P *< 0.05, *P *< 0.01, and *P *< 0.001, respectively

To further explore the mechanisms for oncogenic roles of PKD2 in breast cancer, gene expression profiles of MDA‐MB‐231 with silencing *PKD2* or *PKD2*&*3* were analyzed by using Affymetrix microarrays in duplicates (Figure 4A, Table S10). Upon silencing *PKD2*, 99 genes were upregulated and 185 genes were downregulated (fold change >1.5, *P* < 0.05; Table S11). While silencing *PKD2*&*3*, 89 genes were upregulated and 137 genes were downregulated (fold change >1.5, *P* < 0.05) (Table S11). There were 60 upregulated and 90 downregulated genes only found upon silencing *PKD2*, while 50 upregulated and 36 downregulated genes were only found upon silencing *PKD2*&*3*. There were 39 upregulated and 95 downregulated genes overlapping between silencing *PKD2* and silencing *PKD2*&*3* (Figure 4A‐b and Table S11). To validate the data from Affymetrix assays, mRNA levels of 16 genes were examined by using RT‐qPCR. In MDA‐MB‐231, all of the 16 genes mRNA expression showed the similar changes with the data from Affymetrix assay (Figure 4B). Furthermore, NRAS Proto‐Oncogene (*NRAS*), Transmembrane Protein 19 (*TMEM19*), G Protein Subunit Beta 4 (*GNB‐4*), and Insulin Induced Gene 2 (*INSIG2*) were validated in MDA‐MB‐468 and T47D cells. Upon *PKD2* or *PKD2*&*3 *silencing, mRNA expression of the 4 genes were significantly suppressed as they were in MDA‐MB‐231 cells (Figure 4C). Although the two isoforms, PKD2 and PKD3, shares similar protein structure and oncogenic functions, their downstream targets were not completely the same. For example, *NRAS*, a well‐characterized oncogene, was downregulated upon silencing *PKD2* and silencing both *PKD2* and *PKD3*, but not suppressed when silencing *PKD3* in both MDA‐MB‐231 and MDA‐MB‐468 cells (Figure 4D), which suggested differentially regulatory roles of PKD2 and PKD3 in breast cancer.

NetworkAnalyst was used to explore the network of differentially regulated genes of *PKD2 *and *PKD2*&*3 *in MDA‐MB‐231, Ubiquitin C (UBC) was found to be one of the common hub‐node in the two networks (Figure 4E, Figure S4 and Table S12). Sixty‐two pathways (*P* < 0.05, FDR <0.05) was found enriched through GO analysis of PKD2‐ and PKD2&3‐regulated genes. Sixty‐one of them were commonly found between PKD2 and PKD2&3, where the cell cycle was one of the most significantly commonly enriched pathways (Table S13). In addition, there were 59 pathways are common between PKD2‐ and PKD3‐regulated genes.^20^

### 3.5 Integrated phosphoproteomes and transcriptomes analysis of PKD2 in breast cancer

**Figure 5:**
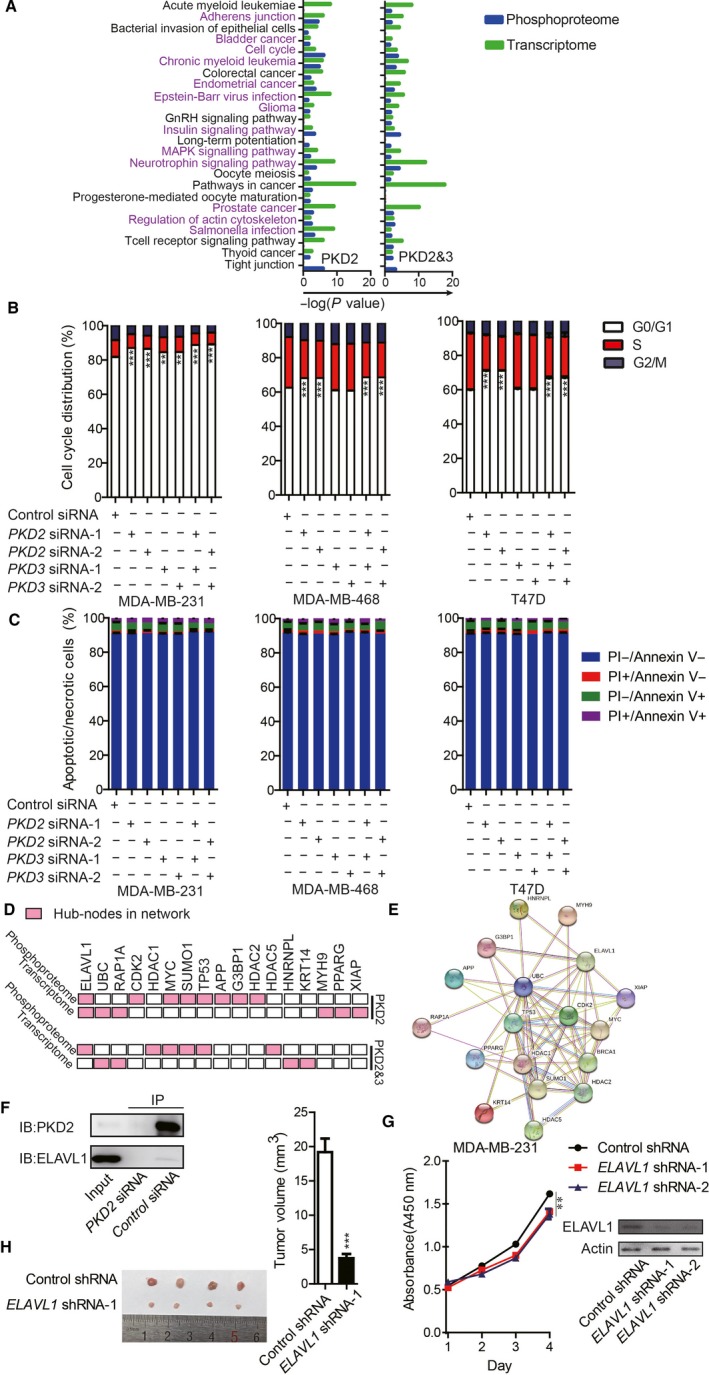
Integrated phosphoproteomes and transcriptomes analysis of PKD2. (A) Analysis of common enriched pathways. (B) Cell cycle and (C) apoptosis analysis on MDA‐MB‐231 upon silencing *PKD2, PKD3* or both *PKD2* and *PKD3*. (D) Integrated analysis of the hub‐nodes of the networks. (E) A network of the hub‐nodes using String. (F) Western blotting showing PKD2 co‐immunoprecipitated with ELAVL1. (G) Silencing *ELAVL1* by *ELAVL1* shRNA inhibited proliferation of MDA‐MB‐231 cells. (H) Silencing *ELAVL1 *by *ELAVL1* shRNA inhibited breast tumor growth in xenograft mouse model using MDA‐MB‐231. Western blot indicated the silencing *ELAVL1* by *ELAVL1* shRNA can effectively knockdown *ELAVL1* in MDA‐MB‐231. We used 4 xenograft mice for control shRNA and 4 xenograft mice for *ELAVL1* shRNA. Palpable tumor growth across time was measured every one week from the time tumor was palpable until the animals were sacrificed (week 3). Tumor images at the end point (week 3) were shown and palpable tumor volumes were measured by width and length with a Vernier caliper and calculated by formula *Volume = (Length × Width × Width)/2*. Error bars represent mean ± SD. The *t*‐test was used for calculation of *P* value. “*,” “**,” and “***” stand for *P *< 0.05, *P *< 0.01, and *P *< 0.001, respectively

Integrated phosphoproteomes and transcriptomes analysis showed there were 24 pathways enriched in at least once in the phosphoproteomes and transcriptomes in MDA‐MB‐231 upon silencing *PKD2* and silencing *PKD2*&*3* (Figure 5A and Table S14). Adherent junction, regulation of actin cytoskeleton, and cell cycle control‐related pathways were also enriched in the similar analysis on PKD3.^20^ More than half enriched pathways (13/24) were common in phosphoproteomes and transcriptomes. Cell cycle analysis showed that silencing *PKD2* had a stronger effects on cell cycle G0/G1 arrest than silencing *PKD3* does (Figure 5B). In addition, no significant effects on cell apoptosis upon silencing PKD2 or silencing both PKD2 and PKD3 (Figure 5C).

The nodes which were top 5 largest number of degree or betweenness were suggested to be the hub‐nodes in the networks. In total, 17 nodes were found to be hub‐nodes in the networks of phosphoproteome and transcriptome (Figure 5D & Table S14). When the network consisting only central nodes was constructed using STRING analysis,^29^ all the central nodes were connected and form a new network (Figure 5E). Six hub‐nodes were shared between the networks of PKD2 and the networks of PKD2&3, and three out of these six central nodes were found as central nodes in the networks of PKD3.^20^

ELAVL1 was found to be the most common center node across PKD2‐ and PKD2&3‐regulated networks from phosphoproteome to transcriptome (Figure 5D). In silico analysis revealed that ELAVL1 contained “LPRTMT “which is a putative PKD consensus motifs (LXRXXS/T). The direct interactions between ELAVL1 and PKD2 or PKD3 were validated in co‐immunoprecipitation (Co‐IP) and mass spectrum (MS) (Figure 5F, Figures S5 and S6 and Table S15). Silencing *ELAVL1* inhibits breast cancer cell growth in vitro and in vivo (Figure 5G,H). Meanwhile, silencing PKD2 in MDA‐MB‐231 led to translocation of ELAVL1 from cytoplasm to nucleus without significant changing ELAVL1 protein level (Figures S5 and S7).

## 4 DISCUSSION

In this study, we found PKD2 and PKD3 were the two major isoforms of PKD overexpressed in breast cancer. Suppressing either PKD2 or PKD3 was able to reduce breast cancer cell proliferation and metastasis. Moreover, inhibition of PKD2 reduced tumor size in vivo. Integrated phosphoproteomes, transcriptomes, and interactome analysis reveals that PKD2 or PKD2&3 regulates multiple cancer‐related pathways. The enrichment of pathway on cell cycle control upon silencing *PKD2* or *PKD2&3* gives a mechanistic explanation to the observation that silencing *PKD2* or *PKD3* inhibits proliferation of breast cancer cell and reduced breast tumor burden. The enrichment of pathways on adherent junction and regulation of actin cytoskeleton gives mechanistic explanations to the observation that silencing *PKD2* or *PKD3* inhibits migration of breast cancer cells.

In addition, we observed enrichment of pathway in angiogenesis upon silencing *PKD2* or *PKD2&3*, supports the current notion that PKD2 or PKD2&3 was involved in tumor blood vessel formation of breast cancer.^30^ Silencing PKD2 or PKD2&3 also led to enrichment of other cancer‐related pathways, for example, insulin signaling pathway, which is documented to play important role in neoplasia via modulating both cancer growth and metastasis.^31^ It's worth to further investigate how PKD2 or PKD2&3 functions in energy metabolism events in breast cancer.

In the PKD2 regulating networks, ELAVL1 was found to be the most common hub‐nodes among the 17 central nodes across PKDs networks from phosphoproteome to transcriptome. ELAVL1, also known as HuR, was proposed to enable multiple cancer phenotypes and have a central tumorigenic activity via selectively binds AU‐rich elements in the 3′ untranslated regions of mRNAs for proto‐oncogenes, cytokines, growth factors, and invasion factors.^32^ Although at current moment, we cannot conclude that oncogenic functions of PKD2 in breast cancer is totally accounted on ELAVL1 due to other identified hub‐nodes in PKD2 regulating networks, our current results at least indicates that oncogenic functions of PKD2 in breast cancer involves ELAVL1 with evidences showing that PKD2 interacts with ELAVL1 and regulates ELAVL1 translocation from cytoplasm to nuclear, and silencing *ELAVL1* shows concrete effects on proliferation of breast cancer cell and breast tumor growth as silencing *PKD2 *does. Besides, Eva Bernhart and his colleges found that RNA‐interference of PKD2 profoundly inhibited growth and changed cell growth of glioma cells. PKD2 knockdown in p53^wt ^glioma cells induced upregulation of p53 and downregulation of the phosphorylation of CDK2.^33^ We have found that TP53 and CDK2 were two central nodes in the PKD2 regulating networks. These researches further suggested that TP53 and CDK2 perhaps play important parts in PKD2 regulating networks of breast cancer. In addition, Qin Hao and his colleges proved that PKD2 or PKD3 knockdown inhibited the phosphorylation of HDAC5 on Ser259 in HCC1806 cells. Our research found that HDAC5 was a center node in PKD2&3 regulating networks.^23^ These studies suggested that HDAC5 may also play a central role in PKD2&3 regulating networks of breast cancer. And according to the research of storz peter and his co‐workers, PKD2 has interaction with PKD3.^34^ This finds reminded that PKD2 and PKD3 perhaps work together to impact breast cancer progression to some degree.

Finally, we found that additive effects of silencing both *PKD2* and *PKD3* compared to silencing *PKD2 *and *PKD3* alone in proliferation and migration of breast cancer cells, and noticeable differences in the outputs of phosphoproteomes and transcriptomes among the breast cancer cells with silencing *PKD2*, *PKD3*, and both *PKD2* and *PKD3 *(Figures 2, 3, 4, 5). In addition, we noticed that PKD2 and PKD3 have common and unique features in their sequences and function domains. PKD2 and PKD3 share a conserved N‐terminal regulatory domain and C‐terminal kinase domain as well as several conserved structural motifs in the regulatory domain, while the intervening sequences between the conserved motifs in the regulatory region are the least conserved.^35^ In addition, PKD2 but not PKD3, contains a N‐terminal hydrophobic stretch of amino acids that potentially insert into the membranes^36^ and a PDZ‐binding motif with C‐terminal autophosphorylation sites.^6^ These results suggested the additive oncogenic functions of PKD2 and PKD3 in breast cancer might due to the isoform‐specific functions of PKD2 and PKD3. The isoform‐specific functions of PKD2 and PKD3 is still waiting to be further characterized.

In conclusion, in the current study, we reported the oncogenic functions of PKD2 and D3 in breast cancer and in regulating cancer‐related pathways to shed light on the oncogenic functions and mechanisms of PKDs in breast cancer.

## CONFLICT OF INTEREST

The authors declare that they have no conflict of interest.
