# Supplementary figures and images for: Oncogenic functions of protein kinase D2 and D3 in regulating multiple cancer‐related pathways in breast cancer
Source: Cancer Med. 2019 Jan 16;8(2):729–41. doi: 10.1002/cam4.1938 (PMC6504119; doi:10.1002/cam4.1938)

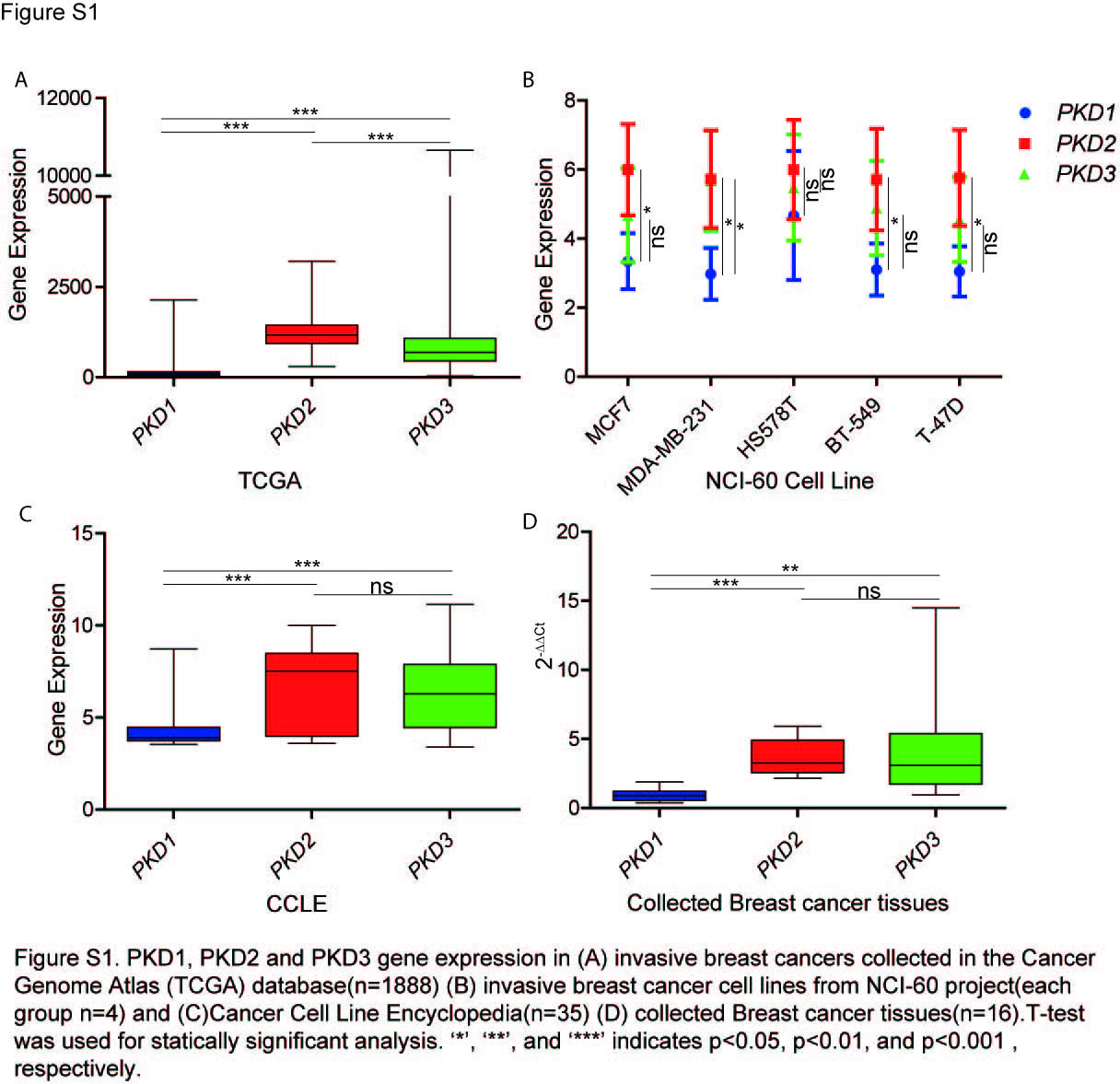

Supplement: Supplementary file 1 [file CAM4-8-729-s001.tif]

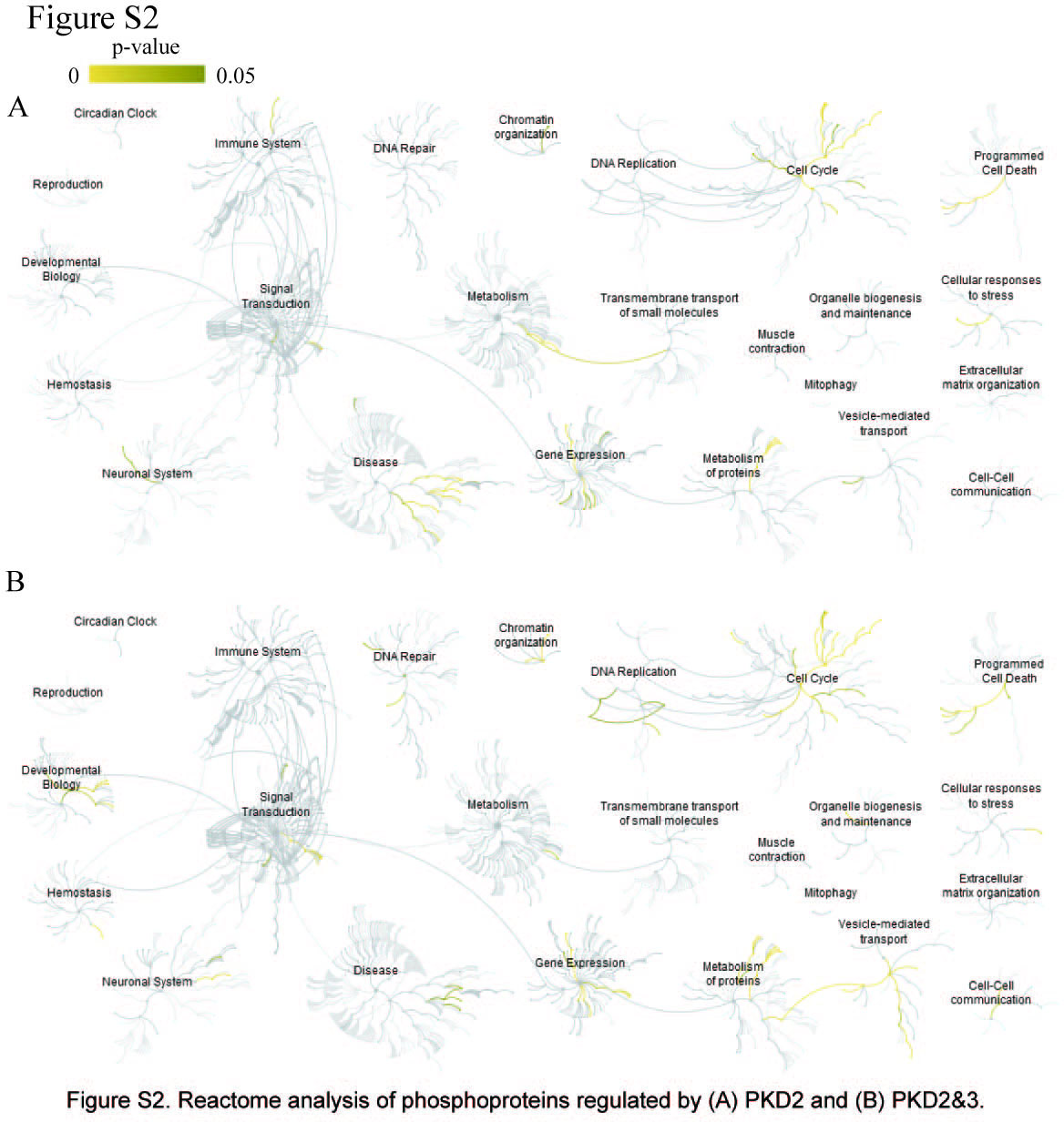

Supplement: Supplementary file 2 [file CAM4-8-729-s002.tif]

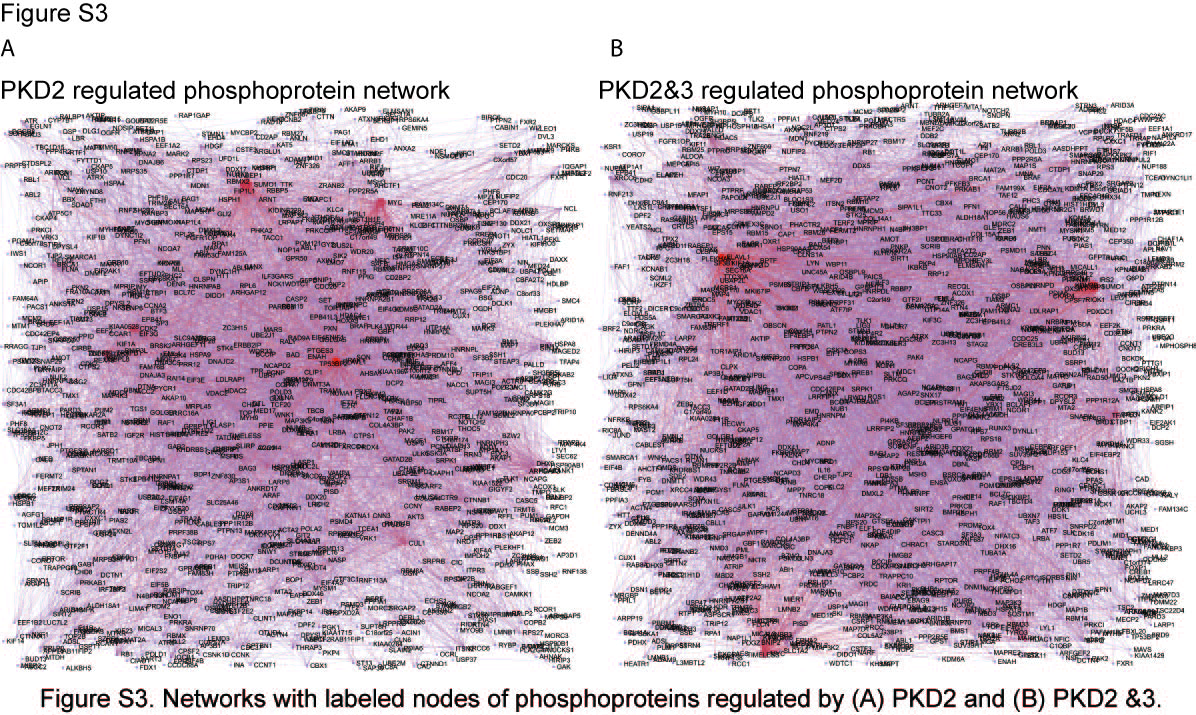

Supplement: Supplementary file 3 [file CAM4-8-729-s003.tif]

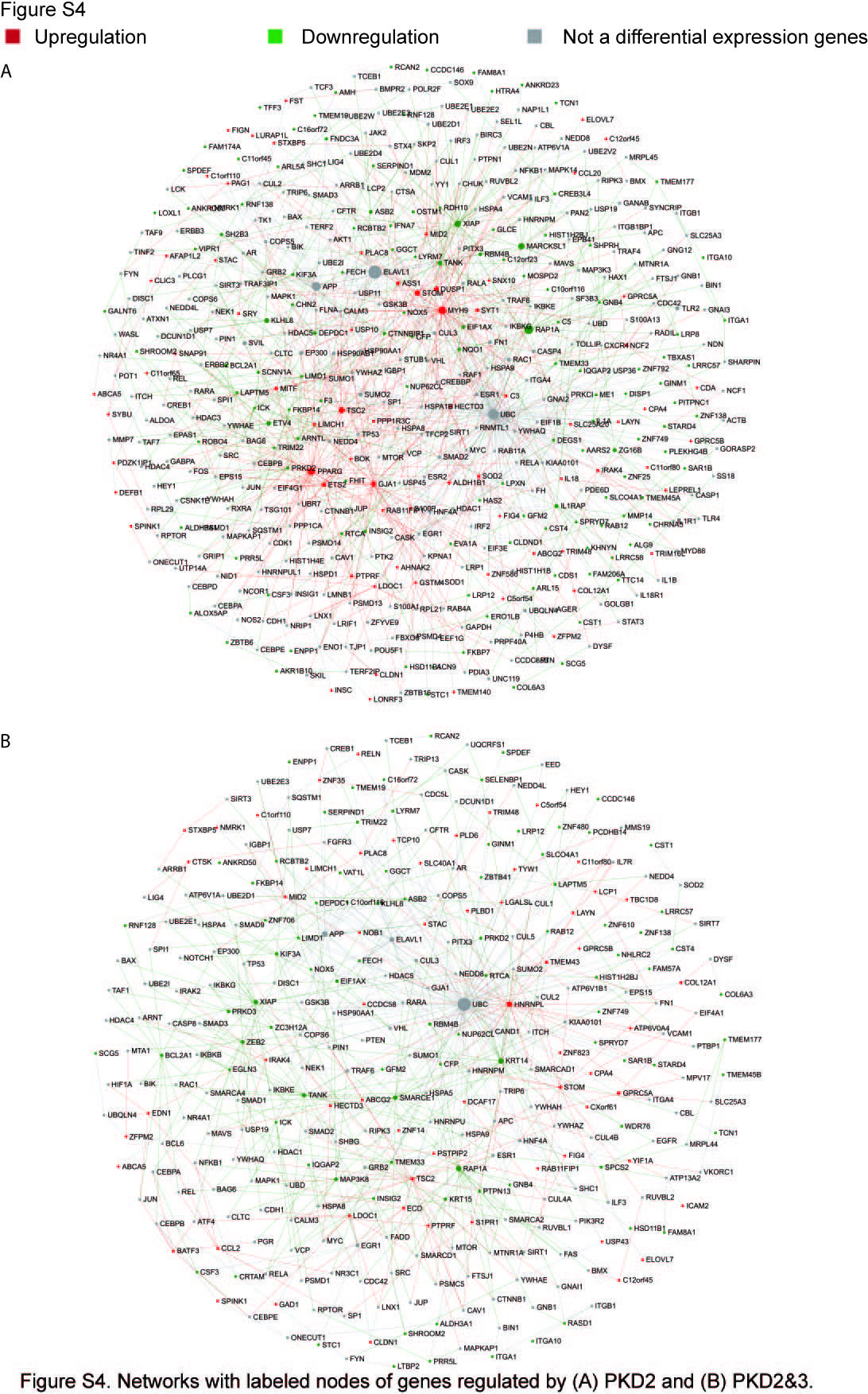

Supplement: Supplementary file 4 [file CAM4-8-729-s004.tif]

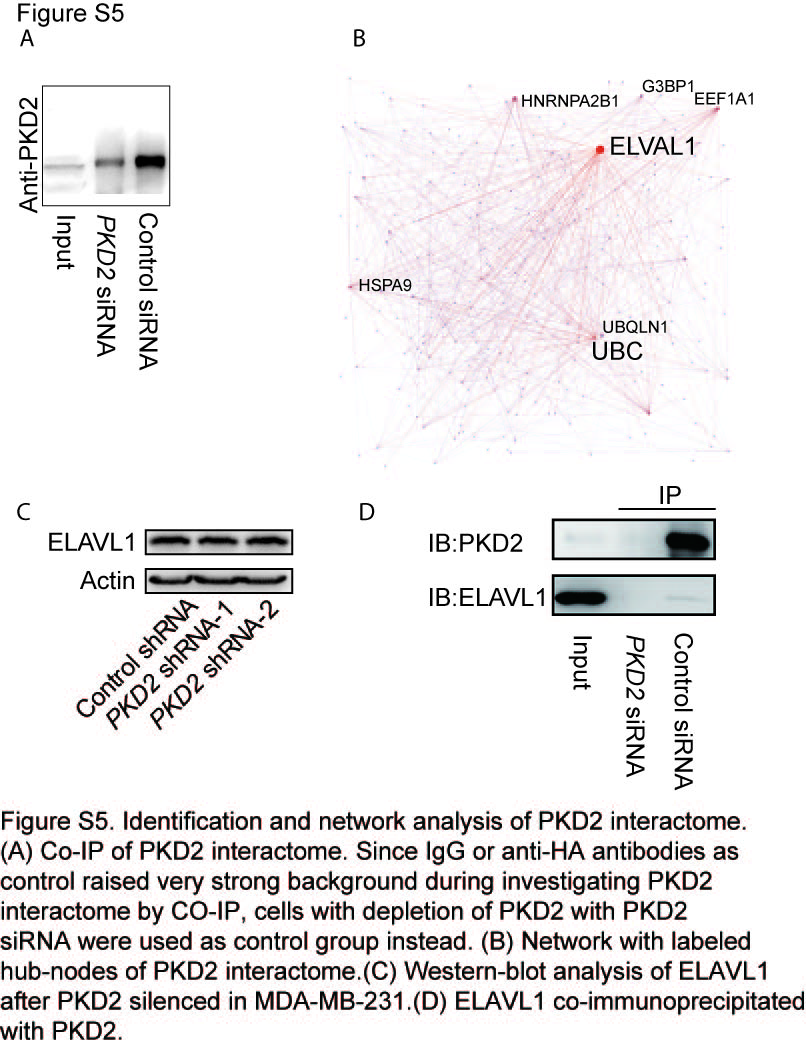

Supplement: Supplementary file 5 [file CAM4-8-729-s005.tif]

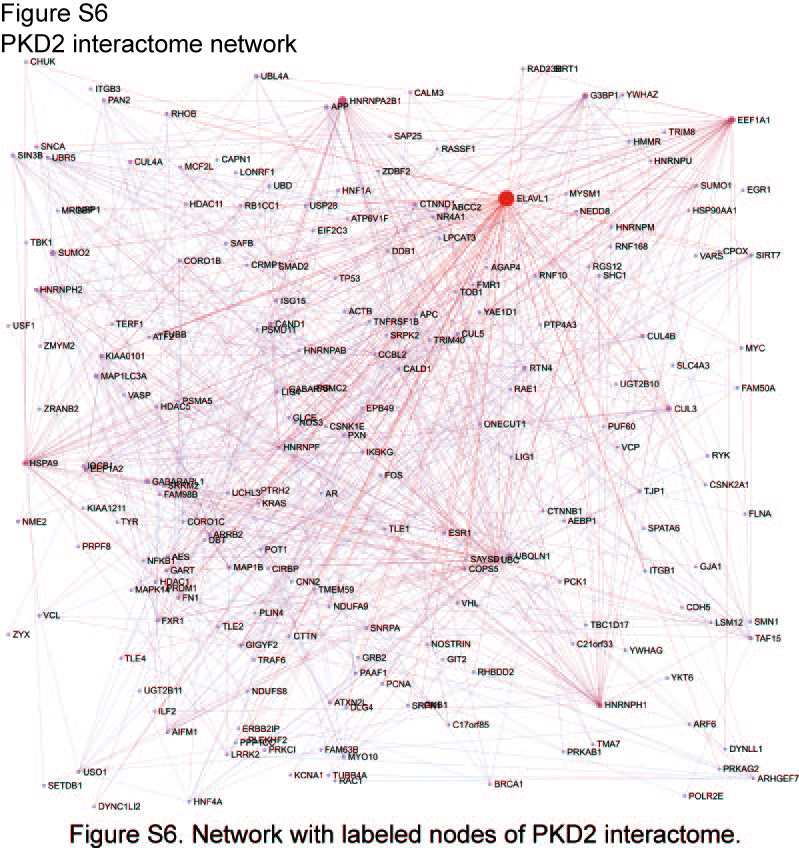

Supplement: Supplementary file 6 [file CAM4-8-729-s006.tif]

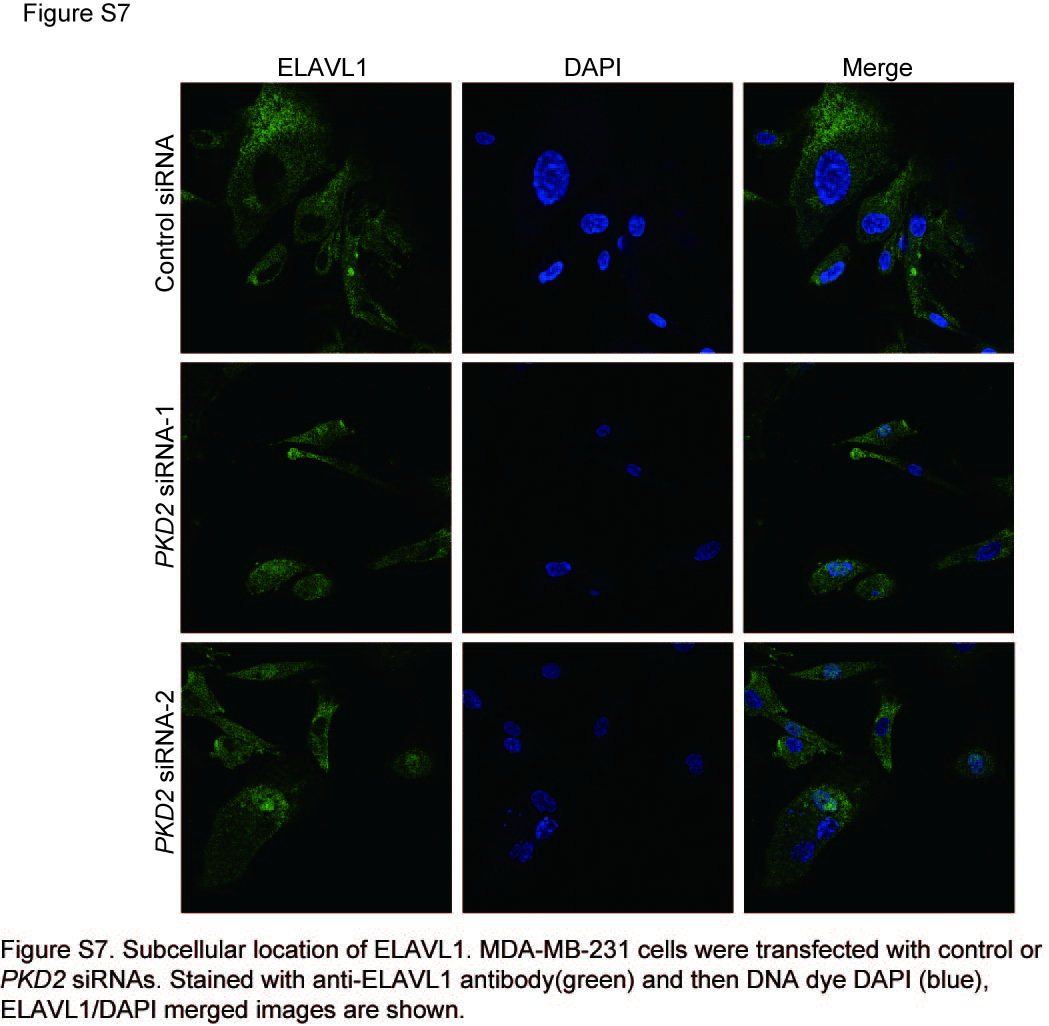

Supplement: Supplementary file 7 [file CAM4-8-729-s007.tif]
